# High-Speed Measurement of Shape and Vibration: Whole-Field Systems for Motion Capture and Vibration Modal Analysis by OPPA Method

**DOI:** 10.3390/s20154263

**Published:** 2020-07-30

**Authors:** Yoshiharu Morimoto

**Affiliations:** 1 4D Sensor Inc., 579-1, Umehara, Wakayama 640-8550, Japan; morimoto@4d-sensor.com; Tel.: +81-(0)73-454-1004; 2 Wakayama University, 930, Sakaedani, Wakayama 640-8510, Japan

**Keywords:** moiré method, grating method, shape measurement, motion capture, vibration analysis, modal analysis

## Abstract

In shape measurement systems using a grating projection method, the phase analysis of a projected grating provides accurate results. The most popular phase analysis method is the phase shifting method, which requires several images for one shape analysis. Therefore, the object must not move during the measurement. The authors previously proposed a new accurate and high-speed shape measurement method, i.e., the one-pitch phase analysis (OPPA) method, which can determine the phase at every point of a single image of an object with a grating projected onto it. In the OPPA optical system, regardless of the distance of the object from the camera, the one-pitch length (number of pixels) on the imaging surface of the camera sensor is always constant. Therefore, brightness data for one pitch at any point of the image can be easily analyzed to determine phase distribution, or shape. This technology will apply to the measurement of objects in motion, including automobiles, robot arms, products on a conveyor belt, and vibrating objects. This paper describes the principle of the OPPA method and example applications for real-time human motion capture and modal analysis of free vibration of a flat cantilever plate after hammering. The results show the usefulness of the OPPA method.

## 1. Introduction

There is a strong demand for high-speed three-dimensional shape measurement; examples include inspection of products on a conveyor belt of a production line, shape measurement performed by moving objects such as automobiles and robot arms, vibration measurement of vibrating objects and soundproofing analysis, and more. Performing full line-inspection instead of sampling inspection can reduce losses due to the manufacturing of defective products. In addition, measurement of the human body is typically difficult because people do not stay still; nursing care, medical care, apparel, sports, and other fields all have real-time shape measurement applications. Regarding high-speed shape measurement, the author previously proposed the integrated phase-shifting method for obtaining the phase of the projected grating in real-time [1]. In addition, as a quantitative analysis method using grating projection, there are many research papers (for example, [2,3,4,5,6]). These methods use the phase shifting method, and an error occurs because it is assumed that movement is stopped during phase shifting. In addition, structured pattern projection methods such as that used by the Kinect [7] rely on the accuracy of the pixel unit. It is less accurate than phase analysis methods, which can analyze at subpixel resolution with high accuracy. There is also an image correlation method [8,9]. Siebert et al. [10] applied this method to the vibration analysis of the membrane. It requires painting random dots on the surface of an object.

Fourier transform profilometry [11,12] also performed phase analysis of a grating pattern with one image, but since a Fourier transformation of the whole grating pattern is performed, it takes a considerable amount of time to calculate, and requires filtering to avoid aliasing. Performing a high-speed measurement with this method can produce large errors, and real-time measurement is difficult.

Yang et al. [13] applied a multiscale image processing method on the frames of the video of a vibrating structure to extract the local pixel phases that encode local modal frequencies, damping ratios, and full-field mode shapes.

A numerical vibration analysis method [14] shows the uses of double-exposure and time averaged moiré fringe projections. However, there are no experimental applications.

The authors proposed the one pitch phase analysis (OPPA) method [15,16,17,18] in which phase analysis of the grating pattern is performed with one image.

The OPPA method is invented based on the moiré topography optical system. Regardless of the distance of the object from the measurement unit, the pitch length (the number of pixels) of the grating on the camera imaging plane is always a constant, so it is possible to extract the brightness data for one pitch at equal intervals and accurately perform the phase analysis. It is possible to eliminate filter processing for aliasing removal. The number of pixels to perform Fourier transformation is small, and fast calculation enables real-time processing. By combining this method with a high-speed camera and performing batch processing, analysis of high-speed phenomena becomes possible.

The principles and application examples of this OPPA method were presented in a paper [17] and proceedings of a few international conferences [15,16,18]. Utilizing the characteristics of the OPPA method, which allows phase analysis of a grating with a single image, it measures the vibration mode of a flat cantilever plate in combination with a high-speed camera [16]. The most commonly used sensor for vibration analysis is the accelerometer [19,20]. However, it is fairly difficult to perform the modal analysis, i.e., to obtain the power and phase distributions on the whole surface of the object. It requires many accelerometers and the procedure is time-consuming. The authors proposed a vibration modal analysis using the OPPA method [16]. As the sensor for the method uses a projector and a camera. The analysis is performed very easily with high accuracy. Furthermore, it was shown that this OPPA method can also be used for the deformation measurement, and it can also be used for incremental measurement of the crack width for diagnosing the degree of deterioration of infrastructure structures such as bridges [18].

This paper is a compilation of the papers including the recent progress of the OPPA method based on the publication of these preprinted manuscripts. As a typical application example of the OPPA method, which is the most important feature, that measures all fields of view at high-speed, as a typical example of motion capture capable of real-time all-pixel three-dimensional coordinate measurement and post-processing, vibration distribution measurement using a high-speed camera. With regard to, an additional explanation will be given by adding new knowledge. For motion capture, examples of arm shape measurement and upper body shape measurement are shown in this paper. 

Regarding vibration measurement, the experiment of vibration modal analysis of a cantilever plate announced by [16] is redone. In this paper, the result of shortening the measurement time will be shown in order to achieve higher speed. The resonance frequencies of 1st to 4th modes are compared with the frequencies obtained by the laser displacement meter, finite element method, and theoretical numerical method.

## 2. Principle of the OPPA Method

In this chapter, the theory of the OPPA method is described by adding the detailed explanation to the reference [17], in order to help understanding of the discussion in later chapters.

### 2.1. Phase Analysis Method

The grating projection method uses a grating to measure the shape (for examples, [1,2,3,4,5,6]), and the sampling moiré method uses a grating to measure deformation [21,22]. The position of the grating is accurately obtained using image processing. By considering the brightness distribution of the grating as a cosinusoidal wave distribution and investigating the phase offset of the cosinusoidal wave at every pixel point, the sampling moiré method has a resolution of about 1/1000 of the grating’s pitch size and performs an analysis with high accuracy. A method of finding the phase angle of this grating is called a phase analysis method.

There are various phase analysis methods (for example, [1,11,21,22,23,24,25]). The author also developed the OPPA method [9,16,17,18]. Here the phase analysis method and the phase-shifting method are described before explaining the OPPA method.

**Figure 1:**
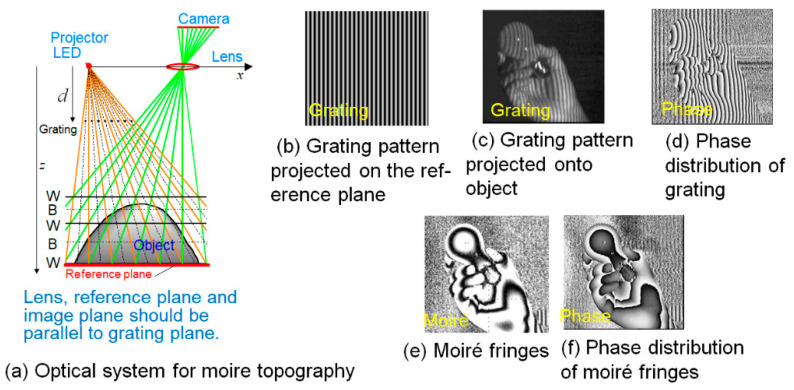
Optical system for shape measurement by the OPPA method [17].

Figure 1a is an optical system of moiré topography, which is one type of grating projection method. Figure 1b shows a grating pattern projected on the reference plane, and Figure 1c shows a grating pattern projected onto the object. The grating lines are distorted according to the height of the object. Figure 1d shows the phase distribution of the grating. Figure 1e shows moiré fringes appearing when the pitch of the projection grating, and the pixel pitch of the camera are equal on the imaging plane. The proof is easily obtained from the geometric similar relationship [17]. Moiré fringes shown in Figure 1e can be also obtained in the same way by thinning out the pixels in Figure 1c (down-sampling). The phase distribution of the moiré fringes is shown in Figure 1f.

**Figure 2:**
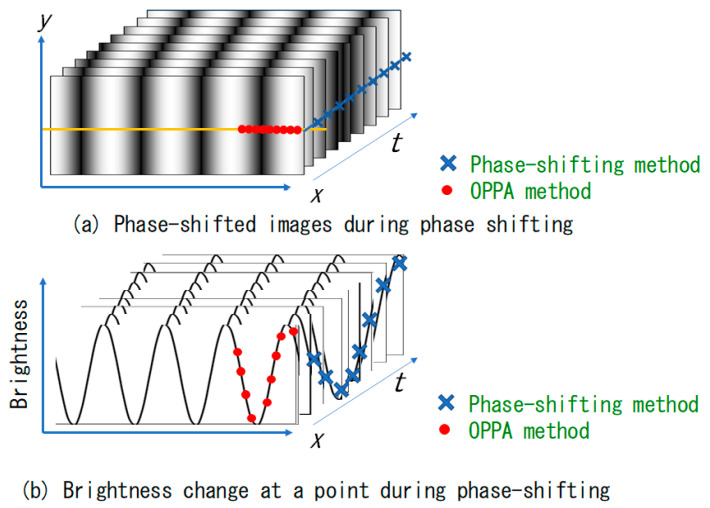
Comparison of phase analysis methods between phase-shifted images and brightness distributions [17].

Figure 2a shows images obtained with equally spaced time increment for one cycle (number of pixels per pitch *N* = 9). The grating images photographed during the shifting of the phase of the grating are displayed. Figure 2b shows the brightness distributions of the central horizontal yellow lines of the grating images.

The blue crosses in Figure 2a,b show the brightness change of the right end point of the central horizontal line. The phase was obtained by analyzing the temporally changing brightness data of the blue crosses during one cycle in the phase-shifting method.

On the other hand, the red circles show the brightness data for one cycle of the space in the first image. A phase analysis can be done with these brightness data. In this paper, the OPPA method used the red brightness data points. Since the first point of blue and the first point (left end) of red are different by just one cycle, they have the same phase value. That is, even if analyzing the blue brightness data or analyzing the red brightness data, the same phase can theoretically be obtained. In the phase-shifting method, since it is analyzed based on only one-point brightness information of the object, the spatial resolution is good, but during a cycle of blue data points, the object must not move. On the other hand, in the case of the OPPA method, since the red point data are obtained with only one image, the time resolution is good. Since it uses the brightness of the *N* pixels of data, the spatial resolution is worse than the phase-shifting method, which uses only one point data.

Even if the brightness distribution is not a cosinusoidal wave form, if the brightness distribution is Fourier transformed and the frequency 1 component is extracted and the inverse Fourier transform is calculated, the resulting brightness becomes a cosinusoidal wave shape. Therefore, whether a cosinusoidal wave is used or a rectangular wave is used, it can be analyzed in the same way.

Note that the phase analysis of the grating in this paper did not directly perform the Fourier transformation. From *N* brightness data *In* (*n* = 1 to *N*) for one pitch, the following Equation (1) or Equation (2) is used to obtain the phase [17]. Equation (1) is the phase of the initial point (first pixel) of successive *N* pixels for one pitch, and Equation (2) is the equation for obtaining the phase of the central point (fifth pixel when *N* = 9) of consecutive *N* pixels for one pitch. In the case of Equation (2), *N* is an odd number.

(1) $$\tan\theta =-\frac{\sum_{n=0}^{N-1}I_{n}\sin\left(n\frac{2\pi }{N}\right)}{\sum_{n=0}^{N-1}I_{n}\cos\left(n\frac{2\pi }{N}\right)}$$

(2) $$\tan\theta =-\frac{\sum_{n=-\left(N-1\right)/2}^{\left(N-1\right)/2}I_{n}\sin\left(n\frac{2\pi }{N}\right)}{\sum_{n=-\left(N-1\right)/2}^{\left(N-1\right)/2}I_{n}\cos\left(n\frac{2\pi }{N}\right)}$$

### 2.2. Shape Measurement by the OPPA Method

**Figure 3:**
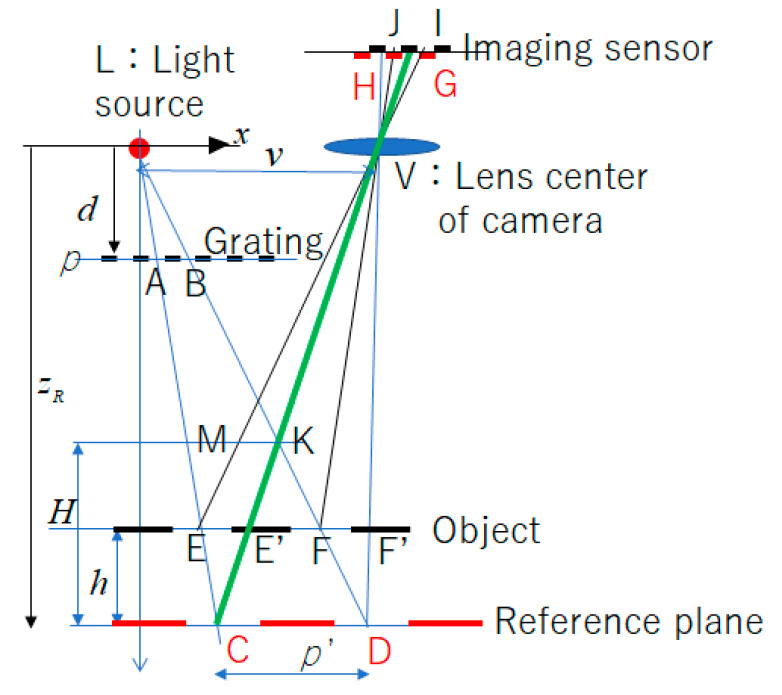
Schematic optical system for explanation [17].

In the case of shape measurement by the OPPA method, the optical system of the moiré topography shown in Figure 1 was used. Figure 3 is an explanatory schematic—a diagram for obtaining the relationship between the phase of the grating and the height of the object. With the position L of the light source as the origin, the *x* axis, the *y* axis, and the *z* axis were measured. The *x* axis and the *y* axis are parallel to the grating plane, the *x* axis is the direction from the light source to the center of the camera lens, the *y* axis is the direction perpendicular to the *x* axis, and the *z* axis is the direction perpendicular to the grating plane. The reference plane is parallel to the grating plane. *p* is the length (AB) of one pitch of the grating on the grating plane, *d* is the height from the light source to the grating plane, *p’* is the length (CD) of one pitch of the grating on the reference plane, *z*_R_ is the height from the reference plane to the light source L, and *v* is the distance (LV) from the light source L to the lens center V of the camera.

In the case of this optical system, as mentioned in Section 2.1, on the imaging plane of the camera, the size of the projected grating’s pitch width is always constant regardless of the height of the measurement surface. The proof was obtained from the following relationship. That is, ∆LAB, and ∆LEF were similar, and ∆VCD and ∆LE’F’ were also similar. Additionally, ∆CEE’ and ∆CLV were similar and also ∆VEF and ∆VIJ were similar. Therefore, the length GH of the image of one pitch length CD on reference plane was the same as the length of IJ of the image of one pitch length EF on object plane. The detailed proof is shown in Reference [17]. Once the number of pixels of the grating for one pitch was adjusted to the integer *N*, even if the height of the object changed, the number of pixels of one pitch in the image of the grating of the object did not change; only the phase of the gating was dependent on the height. By calculating the phase difference between the phase of the object surface and the phase at the reference plane along each pixel’s view-line, the relationship between the phase difference and the height *h* of the object plane from the reference plane is expressed by Equation (3). These relationships can be obtained by considering the simple geometric similarity [9,10].

(3) $$h=\frac{p\Theta Z_{R}{}^{2}}{p\Theta Z_{R}+2\pi vd}=\frac{p^{\prime }\Theta Z_{R}}{p^{\prime }\Theta +2\pi v}$$

where Θ is the phase difference between the phase at the reference plate and the phase at the object on the same pixel of the camera. In the OPPA method, the relationship between phase difference Θ and height *h* is not the function of *x* and *y*, but a function of Θ.

As described above, in the OPPA method, the phase of the grating was analyzed from the brightness data of consecutive *N* pixels in one-shot image.

**Figure 4:**
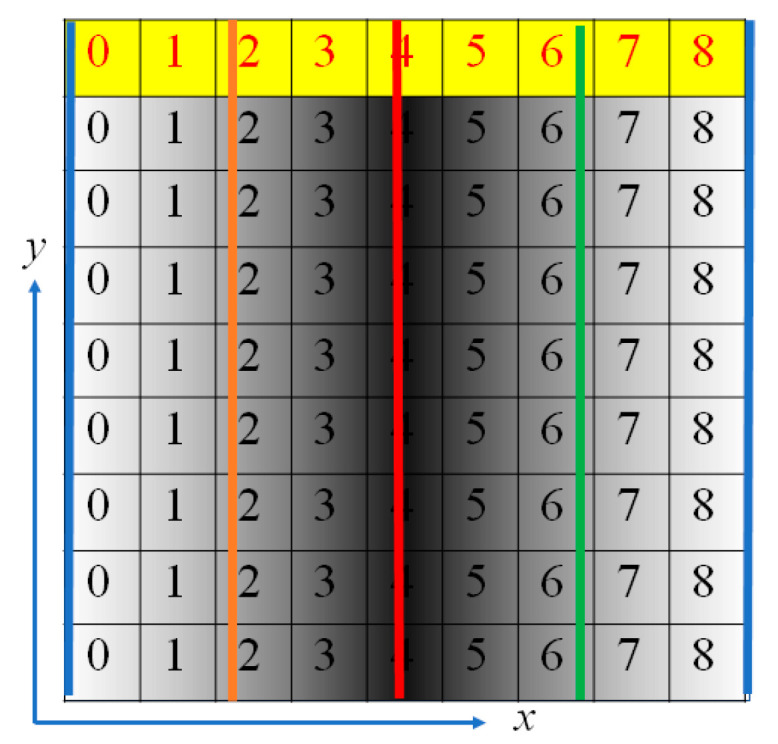
Brightness distribution and phase distribution for OPPA1 [17] (Blue, brown, red and green lines show phases of 0, 90, 180 and 270 degrees, respectively).

Figure 4 is an explanatory view showing one pitch for phase analysis (*N* = 9). Brightness data for *N* pixels on consecutive straight lines in the x-direction were used. This method is called the OPPA 1 method.

**Figure 5:**
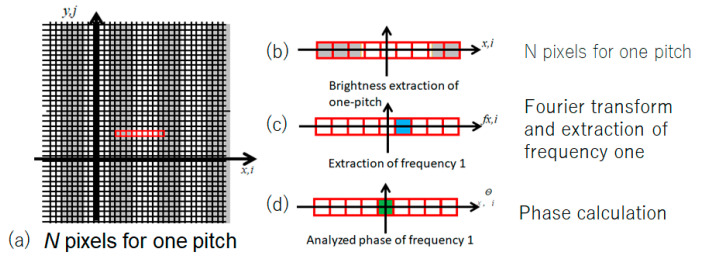
Procedures for OPPA1 [17].

Figure 5 is a schematic diagram showing an example of a grating image photographed by the optical system of Figure 3.

The image data of one pitch (i.e., consecutive nine pixel data) within the red frame in Figure 5a was extracted. As shown in Figure 5b, the Fourier transform of the pixel data was calculated and the frequency components were obtained. The frequency 1 component was extracted as shown in Figure 5c and the other frequencies were filtered as noise, i.e., deleted. The phase at the center of the frequency 1 component was obtained using Equation (2) and was stored as the phase of the central pixel in another memory corresponding to the pixel as shown in Figure 5d. The nine consecutive pixels were shifted pixel by pixel to obtain the phase distribution of all the pixels. From this phase distribution, the phase difference distribution was obtained, and the height *h* distribution was obtained using Equation (3).

### 2.3. Improvement of the OPPA 1 Method

**Figure 6:**
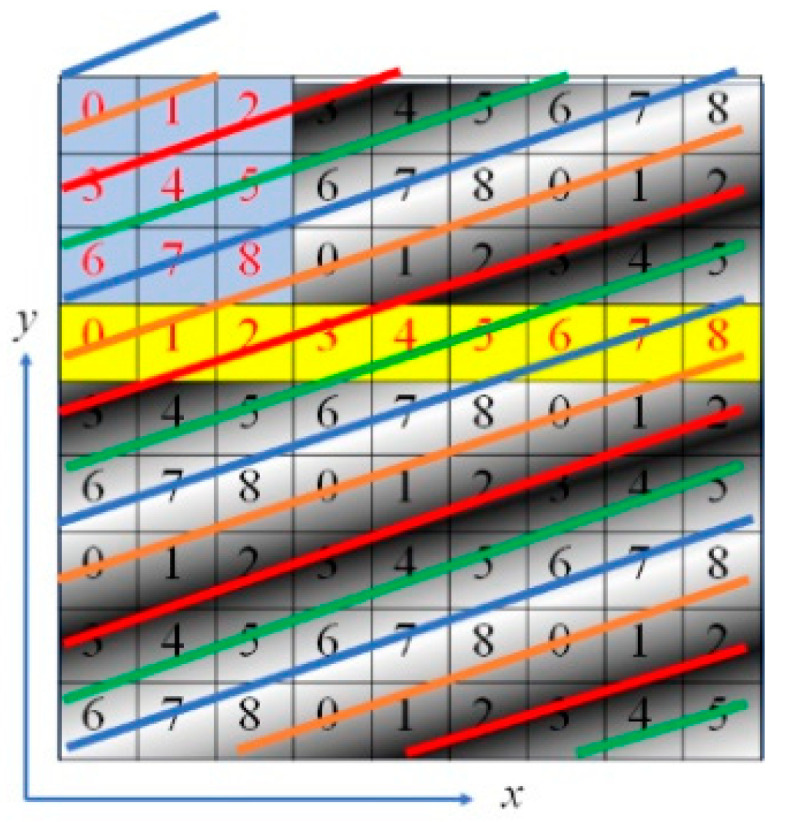
Brightness distribution and phase distribution for OPPA3 [17] (blue, brown, red and green lines show phases of 0, 90, 180 and 270 degrees, respectively.).

In the case of the phase analysis by the OPPA 1 method, as shown in the yellow region of Figure 4, the brightness data were taken from a long and thin region (9 pixels × 1 pixels). We could instead project an oblique grating having an angle *α* (= arctan (⅓)) to the *x*-direction as shown in Figure 6. Then the brightness data of nine pixels in the yellow region were used for phase analysis by OPPA 1. The 3 pixels × 3 pixels in the light blue area in Figure 6 had the same phases as the yellow 9 pixels × 1 pixels. That is, the blue 3 pixel × 3 pixel data can be performed in the same way of analysis using the yellow 9 pixel × 1 pixel data. We call this phase analysis method using 3 pixel × 3 pixel brightness data of an oblique grating as the OPPA 3 method.

Generally, in conventional image processing, when measuring a pixel on an object’s boundary, the pixel views a mix of object region and background region; the result is that this pixel cannot obtain an accurate phase value of the projected grating, therefore, accurate height at this pixel cannot be obtained either. In the case of the OPPA 1 method, the central pixel is five pixels away from the edge. That is, accurate results are not obtained within five pixels of the edge of the object. However, as shown in Figure 6, when the light blue 3 pixels × 3 pixels are used, the central pixel is two pixels away from the edge. That is, accurate results are not obtained within two pixels of the edge of the object. The inaccurate areas of conventional image processing, the OPPA 3 method, and the OPPA 1 method are 1 pixel, 2 pixels and 5 pixels, respectively. Though the OPPA 1 method and the OPPA 3 method are similar, depending on the type of projector and the shape of the object, one method may perform better than the other. In the case of a shape with a lot of rapidly changing areas, the OPPA 3 method may be used, and when there are many areas that can be regarded as a flat surface, the OPPA 1 method may be used.

## 3. Real-Time Motion Capture Using the OPPA Method

Conventional motion capture performs a movement measurement of the human body [26,27] by attaching reflection targets and measuring their coordinates to detect movement. However, the conventional motion capture uses several ball targets at human joints. The number of targets was limited to several dozen points. The ball target positions were not accurate joint positions. There is a lot of research happening around maker less motion capture and capturing of biodata/biometrics, etc. [28,29,30,31]. The author’s system can analyze all pixel points (several hundred thousand pixels) of the camera simultaneously. The analysis is extremely fast and easy.

**Figure 7:**
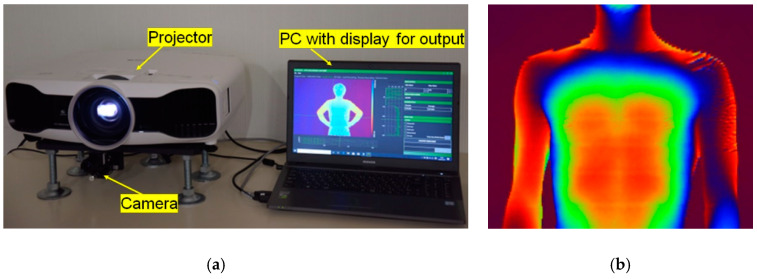
Real-time motion capture system and example of a measured human body. Real-time motion capture system and example of measured human body. (**a**) Real-time motion capture system (OPPA3); (**b**) Measured human body (OPPA1). One of the (Supplementary Materials) is a video of the OPPA Motion Capture.

Figure 7a shows a real-time shape measurement system using the OPPA 3 method. A commercially available high brightness liquid crystal grating projector (EPSON TW8200) was used. A camera was installed under the projector. By using this system, a human body was measured. The measurement result is shown in Figure 7b. In this case, OPPA1 was used. This figure shows an example of the height distribution of a breathing human body. The height distribution is shown in pseudo color at each camera pixel coordinate (*i*, *j*). In this case, *i* and *j* corresponded to the *x* and *y* directions, respectively. Three-dimensional coordinates were obtained at almost all pixel points.

It is difficult for conventional methods of measuring vital capacity to identify the site even if there is an abnormality. However, by measuring the height change of the upper half of the body by using this motion capture, it is possible to identify a lesion with little change. It shows the possibility of a new diagnostic method.

**Figure 8:**
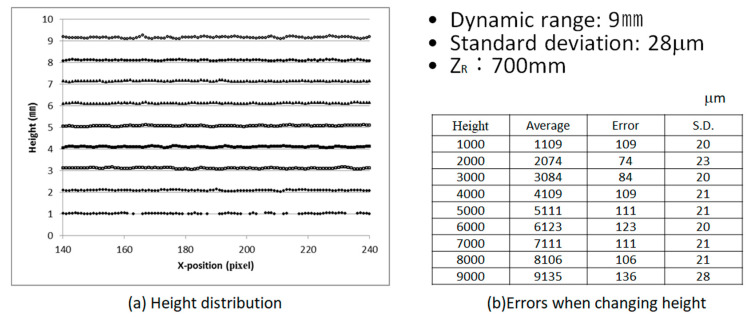
Results of height error and standard deviation of a flat plate.

Figure 8 shows the height error and standard deviation results of measurement of a flat plate when changing the height of the flat plate. In this case the maximum standard deviation was 28 µm. This measurement system’s analyzable area (dynamic range) can be easily changed by altering the distance *z*_R_ in Figure 3 using the same system.

## 4. Measurement of Vibration Distribution by the OPPA Method

It is especially important to measure vibration modes and the frequencies where they occur. The current main method of analysis was performed using accelerometers and measuring many points, or, often requiring the user to change the accelerometer positions many times. The method is introduced as a kind of good textbook [32] and a review [33]. Xiang et al. [19] analyzed the modal analysis of bridge structures. However, since the accelerometer is brought into contact with an object, the vibration characteristics will change. Additionally, also, an analysis using an accelerometer takes a considerable amount of time for experimentation and the analysis is time-consuming. The repeatability of the experiment is not always guaranteed.

In order to analyze the vibration, there are exact formulas describing the observed shift of projected moiré grating lines on an object for the paraxial model [14]. Though it shows the interpretation of fringes produced by time-averaged projection moiré, there is no experimental analysis.

Siebert et al. [10] analyzed the vibration using 3D image correlation. It requires a random dot pattern painted on the object.

As shown in the introduction, the authors previously reported on the vibration analysis of a vibrating membrane by the OPPA1 method [8,9,10] in which phase analysis of the grating pattern is performed with one image. The theoretical treatment, accuracy check and experimental measurement results have been previously shown.

**Figure 9:**
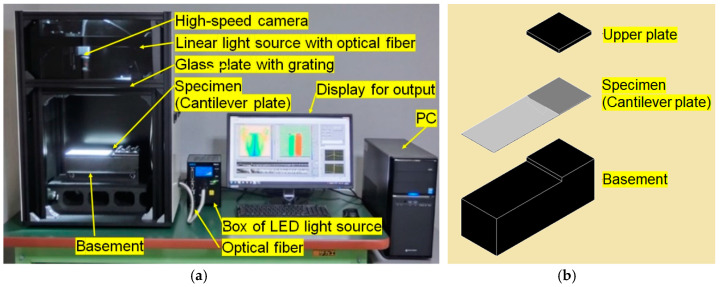
Vibration analysis system using OPPA 1 method. (**a**) Analysis system; (**b**) Specimen (Cantilever plate with heavy basement).

The analysis method was extended to a vibration analysis system capable of vibration mode analysis using a high-speed camera as shown in Figure 9 [16]. As a projector for projecting a grating, a linear light source with 200 μm width and 200 mm length using an optical fiber were developed. By illuminating the glass grating with this linear light source, a large number of grating lines can be projected onto the specimen object. The images of the grating lines were recorded with a high-speed camera, and the time-varying images were analyzed according to the OPPA method to obtain the height distribution of the object. The time change of the height at each pixel was frequency-analyzed to obtain the power distribution and the phase distribution on the entire surface of the object. A flat cantilever plate shown in Figure 9b was used as a specimen. The plate was fixed on one end on a heavy basement with 30 kg. By using this system, the free vibration of a flat cantilever plate after hammering was analyzed. Images were captured for 10 s at 2000 fps, and the height distributions of the 20,000 images were analyzed by the OPPA1 method. The frequency analysis of the height displacement at each pixel of the camera was performed using the Fourier transform. Then the frequency response of the power (squared amplitude) and phase were obtained.

**Figure 10:**
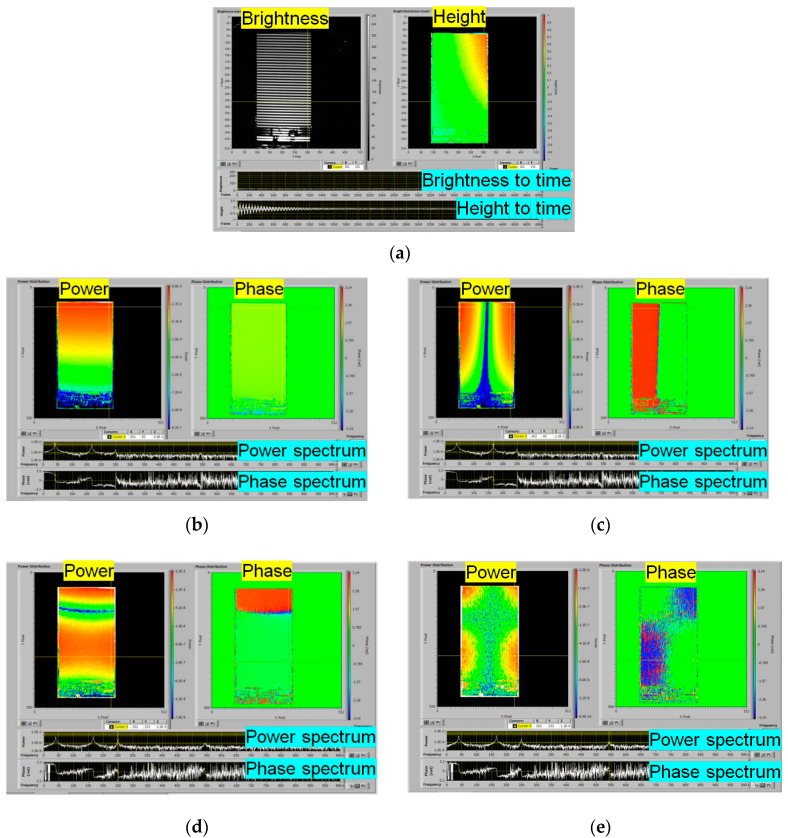
Results of modal analysis of vibrating flat cantilever plate. (**a**) Brightness and height distributions, (**b**) 1st mode (40.0 Hz) (**c**) 2nd mode (166.8 Hz), (**d**) 3rd mode (249.6 Hz) (**e**) 4th mode (543.6 Hz). One of the (Supplementary Materials) is a video of the OPPA Vibration Distribution Analysis.

In this paper, the experiment was performed again. Though the recording speed was 2000 fps, the recording time was 2.5 s. It is shorter than the previous experiment [16]. The resolution of the frequency was 0.4 Hz. The calculation time to obtain the power and phase distributions for all frequencies was 486 s. The calculation time for 10 s [16] was 2504 s. It was shorter than the calculation time for 10 s data. By selecting the resonance frequencies, which had the local maximum power and also at which the phase value changed by 180 degrees, several vibration modes of the surface of the plate could be obtained. Figure 10 shows the results of the modal analysis. The power distribution and the phase distribution for each resonance frequency are displayed in a pseudo color according to the values of the power and phase, respectively. The resonance frequencies of the first, second, third, and fourth-order vibration modes were measured as shown in Table 1.

To evaluate the results obtained by OPPA method, the resonance frequencies were checked with the results using a laser displacement meter, finite element analysis using IronCAD [34] and theoretical calculation [35]. The results of the resonance frequencies are shown in Table 1.

The theoretical *i*-th resonance frequency *f_i_* of a cantilever plate [16] is expressed by the following equation.

(4) $$f_{i}=\frac{\alpha_{i}}{2\pi }\frac{h}{l^{2}}\sqrt{\frac{E}{\rho (1-\nu^{2})}}$$

where *α*_1_ = 1.002, *α*_2_ = 4.310, *α*_3_ = 6.239 and *α*_4_ = 27.28, and *h* is the thickness of the cantilever plate, *l* is the ratio of the length 200 mm in the *x* direction to the length 100 mm in the *y* direction of the plate, *E* is the Young’s modulus, *ρ* is the density, and *ν* is the Poisson’s ratio.

The material of the plate is aluminum, then, *E* = 0.683 × 10^11^ [Pa], *ρ* = 2.7 × 10^3^ [kg/m^3^], *l* = 2, *h* = 0.002 [m] and *ν* = 0.3 are substituted.

The results are shown in Table 1.

**Figure 11:**
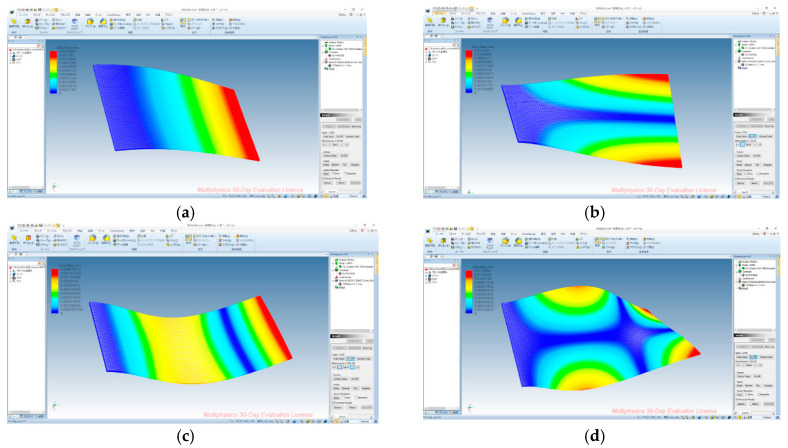
Results of modal analysis using finite element method. (**a**) 1st mode (42.53 Hz); (**b**) 2nd mode (182.08Hz); (**c**) 3rd mode (264.96 Hz); (**d**) 4th mode (592.54 Hz).

The results of the mode analyzed by the finite element method are shown in Figure 11. Though the displacement distributions are displayed with exaggeration, the shapes of the modes were fairly coinciding with each other. The resonance frequencies of the mode were a little higher than the results of other methods.

Table 1 shows that the experimental resonance frequencies almost match the theoretical values. It seems to be even better if more accurate material constants and precise dimensions are selected.

As a result of the OPPA method for measuring vibration, each *i*-th vibration mode is easily measured by experiment with non-contact.

The extraction of resonance frequencies provides accurate amplitude because the extraction is a kind of filtering, which eliminates other frequencies as noise.

In this way, the OPPA vibration analysis system provides a new vibration modal analysis method. The data sampling time is 2.5 s with 2000 fps high-speed camera. The system analyzes about 260,000 points using 512 pixel points by 512 pixel points. This corresponds to 260,000 accelerometers. The measurement method is non-contact and performs simultaneous measurement at all pixels. The accuracy is remarkably high.

## 5. Conclusions

The proposed OPPA method can analyze the phase of the grating projected on an object. The phase can be analyzed with high speed and high accuracy. Additionally, the optical system does not need a phase-shifting device, so system hardware cost is low. By using the OPPA method, a real-time whole surface human motion capture was fabricated. The shape of the upper body of the human body during respiration showed the possibility of analyzing the volume change during respiration from a three-dimensional shape. This makes it possible to identify the defective part of the body.

By implementing with a high-speed camera, a vibration analysis system for the whole surface of the vibrating object was fabricated. It provides a modal frequency analysis of vibrating objects. The free vibration of a flat cantilever plate after hammering was analyzed. By using this system, antivibration measures and noise countermeasures will be simplified. The experimental results using the OPPA method were in good agreement with the results obtained by laser displacement meter, finite element method, and theoretical analysis.

## Figures and Tables

**Table 1:** Comparison of resonance frequencies among OPPA method, laser displacement meter, finite element method and theoretical analysis. (Unit: Hz)

Mode	Experimental		Numerical	
	OPPA Vibration	Laser disp. Meter	FEM	Theoretical
1st	40.2	40.4	42.53	40.4
2nd	167	168.2	182.08	173.78
3rd	249.4	250.8	264.96	251.55
4th	543	549.8	592.54	566.89

## Supplementary Materials

The following are available online at https://www.mdpi.com/1424-8220/20/15/4263/s1.
